# Evidence for an Additive Neurorestorative Effect of Simultaneously Administered CDNF and GDNF in Hemiparkinsonian Rats: Implications for Different Mechanism of Action

**DOI:** 10.1523/ENEURO.0117-16.2017

**Published:** 2017-03-13

**Authors:** Merja H. Voutilainen, Francesca De Lorenzo, Polina Stepanova, Susanne Bäck, Li-Ying Yu, Päivi Lindholm, Eeva Pörsti, Mart Saarma, Pekka T. Männistö, Raimo K. Tuominen

**Affiliations:** 1 Division of Pharmacology and Pharmacotherapy, Faculty of Pharmacy, Viikki Biocenter, University of Helsinki, FIN-00014 Helsinki, Finland; 2 Institute of Biotechnology, Viikki Biocenter, University of Helsinki, FIN-00014 Helsinki, Finland

**Keywords:** 6-OHDA, additive effect, CDNF, ER stress, GDNF, rat

## Abstract

Parkinson’s disease (PD) is a neurodegenerative disorder associated with a progressive loss of dopaminergic (DAergic) neurons of the substantia nigra (SN) and the accumulation of intracellular inclusions containing α-synuclein. Current therapies do not stop the progression of the disease, and the efficacy of these treatments wanes over time. Neurotrophic factors (NTFs) are naturally occurring proteins promoting the survival and differentiation of neurons and the maintenance of neuronal contacts. CDNF (cerebral dopamine NTF) and GDNF (glial cell line-derived NTF) are able to protect DAergic neurons against toxin-induced degeneration in experimental models of PD. Here, we report an additive neurorestorative effect of coadministration of CDNF and GDNF in the unilateral 6-hydroxydopamine (6-OHDA) lesion model of PD in rats. NTFs were given into the striatum four weeks after unilateral intrastriatal injection of 6-OHDA (20 µg). Amphetamine-induced (2.5 mg/kg, i.p.) rotational behavior was measured every two weeks. Number of tyrosine hydroxylase (TH)-positive cells from SN pars compacta (SNpc) and density of TH-positive fibers in the striatum were analyzed at 12 weeks after lesion. CDNF and GDNF alone restored the DAergic function, and one specific dose combination had an additive effect: CDNF (2.5µg) and GDNF (1µg) coadministration led to a stronger trophic effect relative to either of the single treatments alone. The additive effect may indicate different mechanism of action for the NTFs. Indeed, both NTFs activated the survival promoting PI3 kinase (PI3K)-Akt signaling pathway, but only CDNF decreased the expression level of tested endoplasmatic reticulum (ER) stress markers ATF6, glucose-regulated protein 78 (GRP78), and phosphorylation of eukaryotic initiation factor 2α subunit (eIF2α).

## Significance Statement

CDNF [cerebral dopamine neurotrophic factor (NTF)] and GDNF (glial cell line-derived NTF) have shown neuroprotective and neurorestorative effects in rodent and nonhuman primate models of Parkinson’s disease (PD). Here, we show for the first time that defined doses of CDNF and GDNF have an additive effect in restoring dopaminergic (DAergic) function and number of tyrosine hydroxylase (TH)-positive neurons in substantia nigra (SN) of 6-hydroxydopamine (6-OHDA)-lesioned rats. The additive effect suggested different mechanisms of action for the NTFs. Our results indicate that CDNF has a dual action via activation of survival promoting PI3 kinase (PI3K)/Akt and inhibition of endoplasmatic reticulum (ER) stress pathways. CDNF decreased the expression level and activity of ER stress markers *in vitro* and *in vivo*, whereas GDNF had no effect.

## Introduction

Cerebral dopamine neurotrophic factor (NTF) (CDNF) is evolutionarily conserved protein that is located in the endoplasmatic reticulum (ER) (Lindholm et al., 2007; Voutilainen et al., 2015; Lindahl et al., 2017). It is widely expressed in tissues, including the brain (Lindholm et al., 2007). Delivery of CDNF protein or gene therapy with viral vector protects and restores dopaminergic (DAergic) function in rat, mouse and monkey models of Parkinson’s disease (PD) (Lindholm et al., 2007; Airavaara et al., 2011; Voutilainen et al., 2011; Bäck et al., 2013; Ren et al., 2013; Garea-Rodríguez et al., 2016). Thereby CDNF joins the group of NTFs with therapeutic potential in PD.

Glial cell line-derived NTF (GDNF) has well-known effects on both lesioned and intact mature DAergic neurons (Rangasamy et al., 2010; Kordower and Bjorklund, 2013). Through interactions with its GFRα1 coreceptor, GDNF signals mainly via the transmembrane Ret receptor tyrosine kinase, thereby activating MEK/MAPK, PI3 kinase (PI3K)/Akt, Src, and PLCγ pathways, mediating neuronal migration, differentiation, growth, and survival (Airaksinen and Saarma, 2002). While the GDNF signaling pathways are well studied, the ways CDNF exerts its actions are still largely unknown.

Unfolded protein response (UPR) is a homeostatic mechanism, by which cells regulate the volume of protein synthesis and control levels of misfolded/aggregated proteins in the ER. Disturbances in ER homeostasis and/or UPR signaling can result in the induction of prolonged ER stress and trigger cell death (Hetz and Mollereau, 2014). ER stress may contribute to cell death in PD, as it has been indicated in autopsied tissue samples of PD patients, and *in vivo* tissue samples from rodent PD models (Colla et al., 2012). Indeed, intracellular inclusions related to accumulation of misfolded, unfolded or aggregated proteins, such as α-synuclein, is a pathologic hallmark of all forms of PD. Specifically, protein misfolding and aggregation has shown to elicit the ER stress and UPR pathways in both familial and sporadic forms of PD, as well as in several animal models of PD, and provides a potential drug target for developing novel therapies for PD (Voutilainen et al., 2015). It is important to note that MANF (mesencephalic astrocyte-derived NTF)-deficient mice and MANF-deficient *Drosophila* UPR pathways are chronically activated, eventually triggering cell death (Lindahl et al., 2014; Lindström et al., 2016).

Both CDNF and MANF are stable proteins whose structures are different from all other known NTFs (Parkash and Goldman, 2009; Hellman et al., 2011; Latge et al., 2015; Voutilainen et al., 2015). Unlike GDNF, CDNF and MANF have a C-terminally located ER retention signal and therefore a significant amount of these proteins are retained in the ER after translation (Glembotski et al., 2012; Henderson et al., 2013, Mätlik et al., 2015). The expression of MANF is up-regulated in response to ER stress, and MANF deprivation sensitizes cells to ER stress-induced cell death (Apostolou et al., 2008; Tadimalla et al., 2008; Lindahl et al., 2014). Expression of CDNF and MANF rescues neurons from ER stress *in vitro* (Hellman et al., 2011; L. Y. Yu and M. Saarma, unpublished observations). Overexpression of CDNF renders astrocytes less sensitive to ER stress-induced cell damage *in vitro* and reduces the expression and secretion of pro-inflammatory cytokines both in cell culture (Cheng et al., 2013), in the 6-hydroxydopamine (6-OHDA) rat model of PD (Nadella et al., 2014) and in degenerating retina (Neves et al., 2016). Unpublished findings indicate that similarly to brain-derived NTF (BDNF) and GDNF, also CDNF can, via still unknown plasma membrane receptor in ER-stressed cells, activate phosphatidylinositol 3-kinase/a serine/threonine kinase (PI3K/AKT) pathways in cell culture and support the survival of neurons. However, in contrast to other known NTFs, the neurotrophic effect of CDNF *in vitro* can only be observed in ER-stressed or degenerating neurons (K. Krieglstein and M. Saarma, personal communication).

The individual neurotrophic proteins have distinct properties that may be significant to their clinical potential. The striking differences in both structure and function, between CDNF (and MANF) and GDNF, would justify the idea that these proteins could be combined to achieve a greater level of neuroprotection. Therefore, we wanted to test whether a combination of submaximal doses of CDNF and GDNF would have an additive neurorestorative effect in the rat 6-OHDA model of PD compared with injection of either of the trophic factors alone. Since the signaling pathways responsible for mediating the neuroprotective effect of CDNF are still poorly defined, we also compared the ability of CDNF and GDNF to activate MAPK or PI3K/AKT/mechanistic target of rapamycin (mTOR) pathways, and regulate the expression of intracellular ER stress markers, such as ATF6 and glucose-regulated protein-78 (GRP78; also known as BiP), in neuronal cultures and *in vivo*.

## Materials and Methods

### Experimental animals

Male Wistar rats (250-280 g) were housed in groups of three to four under a 12/12 h light/dark cycle at an ambient temperature of 20-23°C. Food pellets (Harlan Teklad Global diet) and tap water were available throughout the experiment *ad libitum*. The animals were housed separately on the surgery days. Animals were returned to home cages 24 h after lesioning. Animals received tramadol 1 mg/ml (Tramal, Orion Pharma) after each surgery session for postoperative pain relief. The experiments were conducted according to the European Community guidelines for the use of experimental animals and approved by the Finnish national Animal Experiment Board. Behavior of the animals was observed on a daily basis and their weight was measured every week.

### Production and purification of human recombinant CDNF and MANF

Human recombinant CDNF was produced and purified as described previously (Lindholm et al., 2007) with an additional thrombin cleavage step to remove tag sequences. GDNF was from PeproTech.

### Administration of 6-OHDA and the NTFs

**Figure 1.**
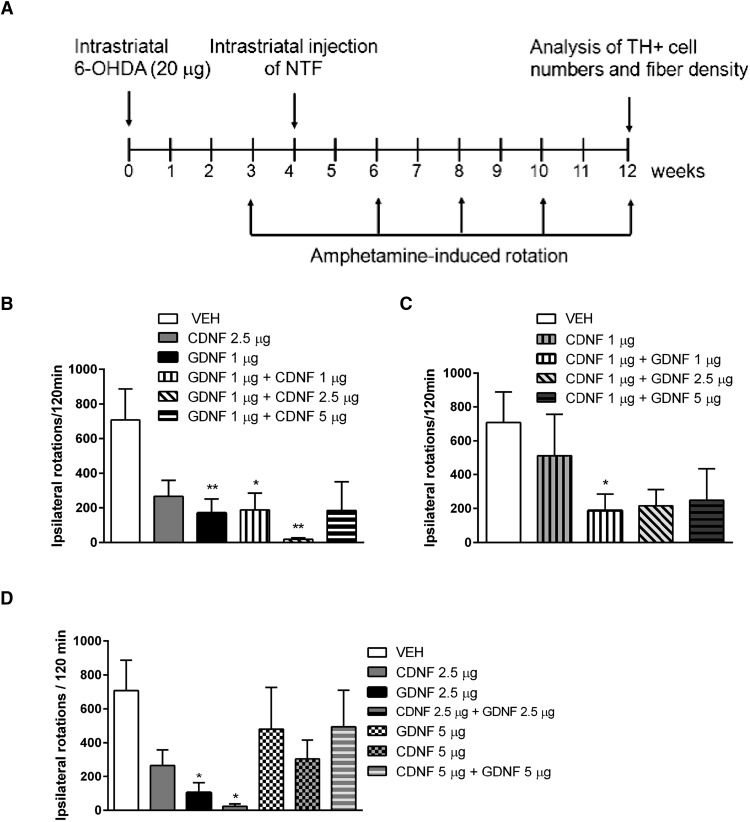
The effect of CDNF and GDNF alone or their coadministration on amphetamine-induced rotation. ***A***, Experimental design. Rats were administered 6-OHDA (20 µg) unilaterally in the striatum. Four weeks later, the rats were given a unilateral intrastriatal injection of CDNF (1 µg), GDNF (1 µg), CDNF (2.5 µg), GDNF (2.5 µg), CDNF (5 µg), or GDNF (5 µg) alone or combination of them. The rotational behavior was measured 3, 6, 8, 10, and 12 weeks after lesion. ***B***, Amphetamine-induced ipsilateral rotations at 12 weeks after lesion in rats treated with vehicle, CDNF (2.5 µg), GDNF (1 µg), or combinations of GDNF (1 µg) with either CDNF (1 µg), CDNF (2.5 µg), or CDNF (5 µg). ***C***, Amphetamine-induced ipsilateral rotations at 12 weeks after lesion in rats treated with vehicle, CDNF (1 µg), alone, or in combination with GDNF (1 µg), GDNF (2.5 µg), or GDNF (5 µg). ***D***, Amphetamine-induced ipsilateral rotations at 12 weeks after lesion in rats treated with vehicle, CDNF (2.5 µg) and GDNF (2.5 µg) alone or in combination; CDNF (5 µg) and GDNF (5 µg) alone or in combination. Means ± SEM are shown; *n* = 8–10 in each group. *p* < 0.05 Tukey/Kramer *post hoc* analysis after one-way ANOVA. *p < 0.05, **p < 0.01.

The design of the experiment was similar to Voutilainen et al. (2009; Fig. 1*A*). Stereotaxic surgery was performed under isoflurane anesthesia (4.5% during induction and 3% during maintenance) as described previously in detail (Voutilainen et al., 2009). The animals received unilateral stereotaxic injections totaling 20 µg of 6-OHDA (Sigma Chemical; calculated as free base and dissolved in ice-cold saline with 0.02% ascorbic acid, 20 µg/4 µl each) in the left striatum using coordinates relative to the bregma and dura (A/P +1, L/M +2.7, D/V –5). At the completion of each injection, the needle was kept in place for 4 min to minimize backflow of the solution. Before the 6-OHDA injections, desipramine (Sigma Chemical; 15 mg/kg, i.p., 1 ml/kg) was administered to prevent the uptake of 6-OHDA into noradrenergic nerve endings. Four weeks later, the rats were given a unilateral intrastriatal injection of CDNF (1 µg), GDNF (1 µg), CDNF (2.5 µg), GDNF (2.5 µg), CDNF (5 µg), or GDNF (5 µg) alone or combination of them (in 4 µl of PBS) into the same site.

### Behavioral analysis

Behavioral tests were conducted one week before (three weeks after 6-OHDA injection) and two, four, six, and eight weeks after NTF-infusion as described previously (Lindholm et al., 2007; Voutilainen et al., 2009). Drug-induced rotational behavior was measured in automatic rotometer bowls (Med Associates). Following a habituation period of 30 min, a single dose of D-amphetamine (2.5 mg/kg, Division of Pharmaceutical Chemistry and Technology, University of Helsinki) was injected intraperitoneally. The rotation sensor recorded full (360°) clockwise and counterclockwise uninterrupted turns for a period of 2 h, and ipsilateral rotations were assigned a positive value.

### Methods applying immunohistochemistry

Transcardial perfusion and tissue processing were done as described previously (Voutilainen et al., 2009). Free-floating sections were processed for tyrosine hydroxylase (TH)-immunohistochemistry as previously described (Voutilainen et al., 2009). In addition phospho (p)S6-, pAkt-, and pERK-immunohistochemistry was conducted as described previously in Iherman-Hella et al. (2014). Stereologic counting of the number of TH-positive cells in the substantia nigra pars compacta (SNpc) was done using the optical fractionator method in combination with the dissector principle and unbiased counting rules (West et al., 1991; Lindholm et al., 2007; Voutilainen et al., 2009). The optical densities (ODs) of the TH-positive fibers and of pS6- and pAkt in the striatum were determined using three coronal striatal sections from each rat as previously described (Voutilainen et al., 2009). The analysis was performed under blinded conditions and the data are presented as the percentage of the intact side defined as 100%.

### Analysis of TH and DAT levels in NTF-treated brain

Forty rats received a single injection of 6-OHDA (20 µg/4 µl) into the left striatum, and an injection of 0.02% ascorbic acid (4 µl, sham-lesion) into the right striatum (A/P +1.0; L/M ±2.7; D/V -5.0, according to bregma). Four weeks later vehicle (PBS), GDNF (1 µg), CDNF (2.5 µg), or GDNF (1 µg) and CDNF (2.5 µg) together (4 µl/1 µl/min) was injected bilaterally according to the same coordinates as above. After an additional four weeks rats were decapitated and the rat striatum (3-mm punch from 2-mm section of each side) and SN (2-mm punch from 2-mm section of each side) were dissected out and stored in -80°C until analysis by Western blotting.

### Western blotting

Striatal and nigral samples were homogenized in 300 μl of lysis buffer (5 mM HEPES, pH 7.4; 320 mM sucrose; 1 mM EDTA; 0.1% SDS; and protease inhibitors; Roche) using a sonicator (Rinco Ultrasonics). Following incubation on ice for 30 min, the homogenates were centrifuged at 1000 × *g* for 10 min (4°C), and protein concentrations in the supernatants were determined using a BSA kit (Pierce).

Samples were further diluted in lysis buffer and Laemmli buffer containing 2% mercaptoethanol and 20 µg of protein was loaded on a 10% SDS-polyacrylamide gel. After transfer of proteins to a nitrocellulose membrane, the membrane was incubated in 5% BSA for 1.5 h to block unspecific binding and then incubated in the presence of mouse anti-TH (1:5000, MAB 318, Millipore), goat anti-DAT (1:1000, sc2020, Santa Cruz Biotechnology), mouse anti-β-actin (1:2000, A1978, Sigma-Aldrich), rabbit anti-pERK (1:1000, 9101, Cell Signaling), rabbit anti-ERK (1:1000, 9102, Cell Signaling), rabbit anti-pAkt (1:2000, 9271, Cell Signaling), rabbit anti-Akt (1:1000, 9272, Cell Signaling), rabbit anti-p-eukaryotic initiation factor 2α subunit (eIF2α) (1:1000, 3398, Cell Signaling), rabbit anti-eIF2α (1:1000, 9722, Cell Signaling), rabbit anti-GRP78 (1:1000, sc-1051, Santa Cruz Biotechnology), and mouse anti-β-tubuline antibodies at 4°C overnight. Membranes were further incubated in horseradish peroxidase-conjugated secondary antibodies [goat anti-mouse 1:2000 (R&D Systems), donkey anti-goat 1:1000 (Santa Cruz Biotechnology), or donkey anti-rabbit 1:3000 (Santa Cruz Biotechnology)], and protein bands were detected using a chemiluminescent substrate (Pierce) with GeneGnome chemiluminescent detector (Synoptics). Quantification of the detected bands was done using ImageJ software version 1.43u (NIH). All results are given as relative density as compared with the loading control (β-actin or β-tubulin) and to an internal control (sample pooled from four intact rats).

### Primary cultures of embryonic dopamine neurons

The midbrain floors were dissected from the ventral mesencephali of 13-d-old NMRI strain mouse embryos. Tissues were incubated with 0.5% trypsin (ICN Biomedical) in HBSS (Ca^2+^/Mg^2+^ free) (Invitrogen) for 20 min at 37°C, then mechanically dissociated. The neurons were cultured 5-7 d with GDNF (50 ng/ml; Icosagen). Then the cultures were treated with thapsigargin (200 nM) (Invitrogen). CDNF (100 ng/ml), GDNF (50 ng/ml), or their combination was added to the cultures at the same time. After 24 h, RNA from cultured cells was isolated by TriReagent (Molecular Research Center) according to manufacturer’s instructions. RNA was reverse transcribed to cDNA with RevertAid Premium Reverse Transcriptase (Fermentas UAB, Thermo Fisher Scientific). Quantitative PCR was performed using LightCycler 480 SYBR Green I Master (Roche Diagnostics GmbH) and Roche LightCycler 480 Real-Time PCR System. The expression levels were normalized to the levels of β-actin in the same samples. Primers used in quantitative PCR were synthetized using previously published sequences (Lindahl et al., 2014).

### Statistical analysis

Data were analyzed using one-way ANOVA (or one-way ANOVA for repeated measures in behavioral studies) followed by Tukey/Kramer’s *post hoc* test. Results are expressed as mean ± SEM and considered significant at *p* < 0.05.

## Results

### Effect of trophic factors on rotational behavior in hemiparkinsonian rats

Animals were first injected with 6-OHDA (20 μg) and four weeks later with the trophic proteins or corresponding vehicle (neurorestoration paradigm; Fig. 1*A*). Amphetamine (2.5 mg/kg, i.p.)-induced ipsilateral turning behavior indicates compromised function of midbrain DAergic neurons in the lesioned side of the brain (Fig. 1*B–D*). In dose-finding studies, it became clear that high doses (5 or 10 µg per single intrastriatal injection) of either CDNF or GDNF alone caused near maximal neurorestorative effect that could not be modified by combining the two proteins (data not shown). Therefore, we tested smaller doses as follows: GDNF (1 µg) alone and in combination with CDNF (1, 2.5, or 5 µg); CDNF (1 µg) alone and in combination with GDNF (1, 2.5, or 5 µg; Fig. 1*B*,*C*). Finally, we also compared the effect of CDNF (2.5 µg) + GDNF (2.5 µg) with either CDNF (5 µg) or GDNF (5 µg) alone (Fig. 1*D*). The only significantly additive neurorestorative effect was observed after combination of CDNF (2.5 µg) and GDNF (1 µg) (Fig. 1*B*), and these doses were used for further studies.

The rotations were significantly reduced in animals treated with GDNF (1 µg) alone, or with the combination of GDNF (1 µg) + CDNF (2.5 µg) when quantified 12 weeks (1B) after lesion (Fig. 1*B–D*, treatment effect: *F*_(3,27)_ = 4.799, *p* = 0.008, *n* = 7-9 per group; Table 1, a). *Post hoc* analyses showed statistical differences at 10 weeks after lesion between vehicle and GDNF (1 µg) group (*p* = 0.004) and between vehicle and CDNF (2.5 µg) + GDNF (1 µg) group, *p* = 0.001; Table 1, b–d) and also at 12 weeks after lesion between vehicle and GDNF (1 µg) group (*p* = 0.006), vehicle and CDNF (2.5 µg) (*p* = 0.029) and between CDNF (2.5 µg) + GDNF (1 µg) group (*p* = 0.001; Table 1, e–g). Combination of GDNF (1 µg) and CDNF (1 µg) significantly reduced the amphetamine-induced rotations at 10 weeks, and at 12 weeks after lesion (Fig. 1*B*).

**Table 1:** Statistical table

Symbol in text	Dataset	Data structure	Type of test	*p* value
a	Fig. 1B, ipsilateral rotations at 12 weeks: GDNF (1 µg) vs PBS at 12 weeks	Normal distribution	One-way ANOVA, Tukey	0.006
b	Fig. 1B, ipsilateral rotations at 12 weeks: GDNF (1 µg) + CDNF (1 µg) vs PBS at 12 weeks	Normal distribution	One-way ANOVA, Tukey	0.0306
c	Fig. 1B, ipsilateral rotations at 12 weeks, GDNF (1 µg) + CDNF (2.5 µg) vs PBS at 12 weeks	Normal distribution	One-way ANOVA, Tukey	0.001
d	Fig. 1B, ipsilateral rotations at 10 weeks: CDNF (2.5 µg) vs PBS at 12 weeks	Normal distribution	One-way ANOVA, Tukey	0.029
e	Fig. 1D, ipsilateral rotations at 12 weeks, GDNF (2.5 µg) vs PBS at 12 weeks after lesion	Normal distribution	One-way ANOVA, Tukey	0.0104
f	Fig. 1D, ipsilateral rotations at 12 weeks, GDNF (2.5 µg) + CDNF (2.5 µg) vs PBS at 12 weeks after lesion	Normal distribution	One-way ANOVA, Tukey	0.0113
g	Fig. 2A, TH cell numbers, GDNF (1 µg) + GDNF (2.5 µg) vs VEH	Normal distribution	One-way ANOVA, Tukey	0.046
h	Fig. 2B, Nissl-positive cells, All NTF-treated groups differed from VEH	Normal distribution	One-way ANOVA, Tukey	0.0001
i	Fig. 4C, OD of pS6 staining in STR. CDNF (2.5 µg) vs GDNF (1 µg)	Normal distribution	One-way ANOVA, Tukey	0.0184
j	Fig. 4D, pERK-positive cells in STR. All NTF-treated groups differed from VEH	Normal distribution	One-way ANOVA, Tukey	0.0077
k	Fig. 5A, level of ATF6 expression in DA neuron cultures, CDNF (100 ng/ml) vs control, no factor	Normal distribution	One-way ANOVA, Tukey	0.04
l	Fig. 5A, level of ATF6 expression in DA neuron cultures, combination [CDNF (100 ng/ml) + GDNF (50 ng/ml)] vs control	Normal distribution	One-way ANOVA, Tukey	0.0102
m	Fig. 5E, GRP78 expression, CDNF (2.5 µg) vs GDNF (1 µg)	Normal distribution	One-way ANOVA, Tukey	0.014

### Effect of trophic factors on DA neurons in the SN of hemiparkinsonian rats

**Figure 2.**
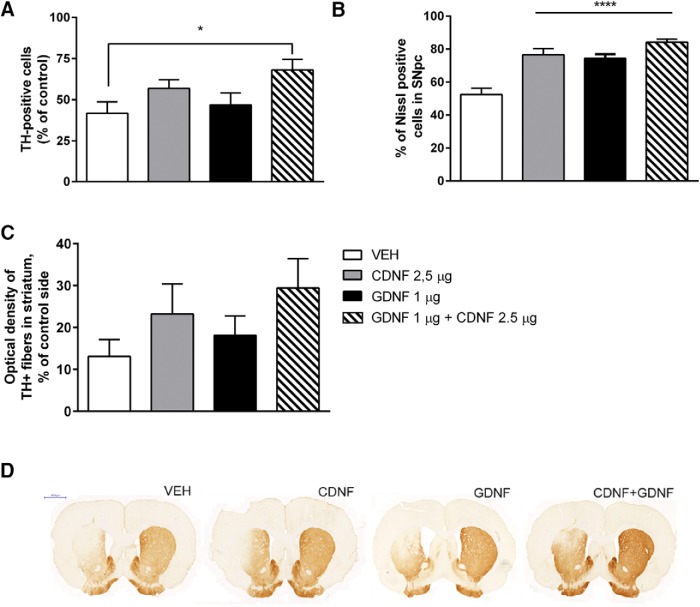
Number of TH-positive and Nissl-positive cells in the SNpc and OD of TH-positive fibers in the STR. ***A***, The effect of CDNF (2.5 μg) alone and GDNF (1 μg) alone, or the combination of CDNF (2.5 μg) + GDNF (1 μg) on TH-positive cell bodies in the SNpc analyzed at 12 weeks after lesion. The effect of the combination of CDNF (2.5 μg) and GDNF (1 μg) differed significantly from the vehicle-treated group (*p* < 0.05). ***B***, Analyses of Nissl-stained cell bodies in SNpc. Results were in line with TH-positive cell body counts from SNpc. ***C***, The effect of CDNF (2.5 μg) alone, GDNF (1 μg) alone, or the combination of CDNF (2.5 μg) and GDNF (1 μg) on TH-positive fibers in the STR. CDNF and the combination of CDNF and GDNF partly rescue TH-positive fibers in the STR. ***D*,** Representative TH-stained striatal sections for each treatment. Mean ± SEM in (***A***, ***B***) *n* = 7-12 and in (***C***) *n* = 7-12 in each group. Tukey/Kramer *post hoc* analysis after one-way ANOVA. *p < 0.05, ****p < 0.0001.

At four weeks after the 6-OHDA injection the loss of DAergic cells in the SN was almost fully developed as in the lesion-control group, the number of TH-positive cells in the lesion side was 55% of that in the intact side (100%; data not shown). In the vehicle-treated group, the number of TH-positive cells was 42% when measured 12 weeks after 6-OHDA (Fig. 2*A*). Thus, there was an additional 13% reduction in TH-positive cell number. In comparison, in rats treated with GDNF (1 µg), CDNF (2.5 µg), or their combination the corresponding numbers were 47%, 53%, and 67%, respectively (Fig. 2*A*) indicating protection and recovery of the TH-positive neurons from 6-OHDA-induced cell death. The effect of the combination of CDNF (2.5 µg) + GDNF (1 µg) differed significantly from the vehicle-treated group (treatment effect: *F*_(3,33)_ = 2.830, *p* = 0.055, *n* = 7-12 per group; Table 1, h). *Post hoc* analyses showed statistical differences between vehicle and GDNF (1 µg) + CDNF (2.5 µg) group (*p* = 0.046). There was no statistical difference between groups treated with single protein and vehicle (Fig. 2*A*). The number of Nissl-stained cells in SNpc correlated well with TH-positive cell numbers in SNpc (Fig. 2*B*). There was a statistically significant difference in Nissl stained cells in rats treated with GDNF (1 µg), CDNF (2.5 µg), or their combination versus vehicle-treated rats.

### Effect of trophic factors on TH-positive fiber density in the striatum of hemiparkinsonian rats

OD of TH-positive fibers was measured to assess whether the trophic factor injection was able to induce regeneration and/or sprouting of fibers. Administration of CDNF (2.5 µg) alone or coadministration of CDNF (2.5 µg) + GDNF (1 µg) showed tendency for a protective effect, but there were no statistical differences (23% and 29% of the intact side, respectively) as compared with the vehicle-treated group (13% of the intact side). Neither GDNF (1 µg) alone had significant effect on TH-positive fibers (18%; Fig. 2*B*,*C*).

### CDNF exerts its effects independently of ERK in naíve rats and in hemiparkinsonian rats

**Figure 3.**
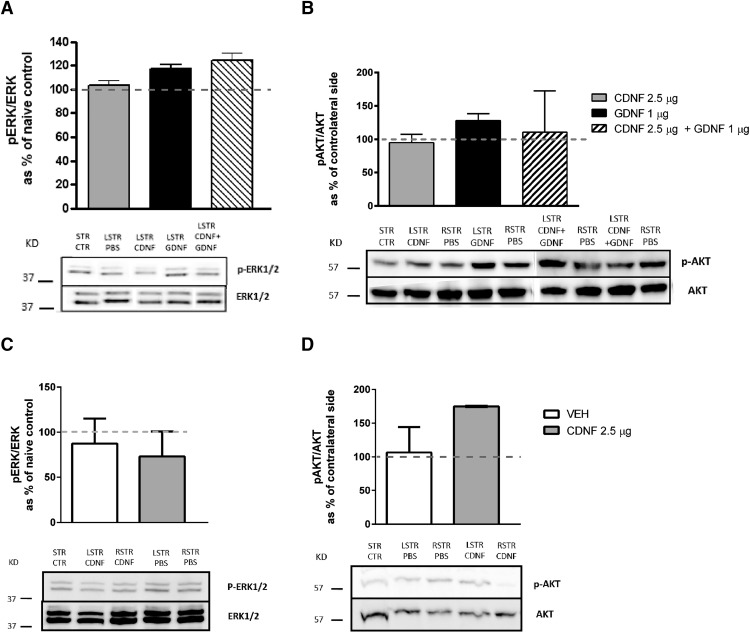
Different effects of CDNF and GDNF on ERK1/2 and PI3K/AKT pathways in naïve rats. Ability of CDNF and GDNF to activate MAPK or PI3K/AKT/mTOR pathways was studied injecting CDNF (2.5 µg), GDNF (1 µg), or their combination into the left STR (LSTR) of naïve rats (***A–D***). Sham-operated rats received PBS into LSTR. ***A***, ***B***, In rats dissected 1 h after NTF injection, GDNF activated ERK1/2 and PI3K/AKT pathway whereas CDNF had no effect. ***D***, In rats dissected 4 h after CDNF injection the PI3K/AKT pathway was activated in nonlesioned brains (***D***), whereas the ERK1/2 pathway was not affected (***C***). Representative Western blot images of ERK1/2, p-ERK1/2, PI3K/AKT, and p-PI3K/AKT are shown. Mean ± SEM, *n* = 3-6 in each group. Tukey/Kramer *post hoc* analysis after one-way ANOVA.

**Figure 4.**
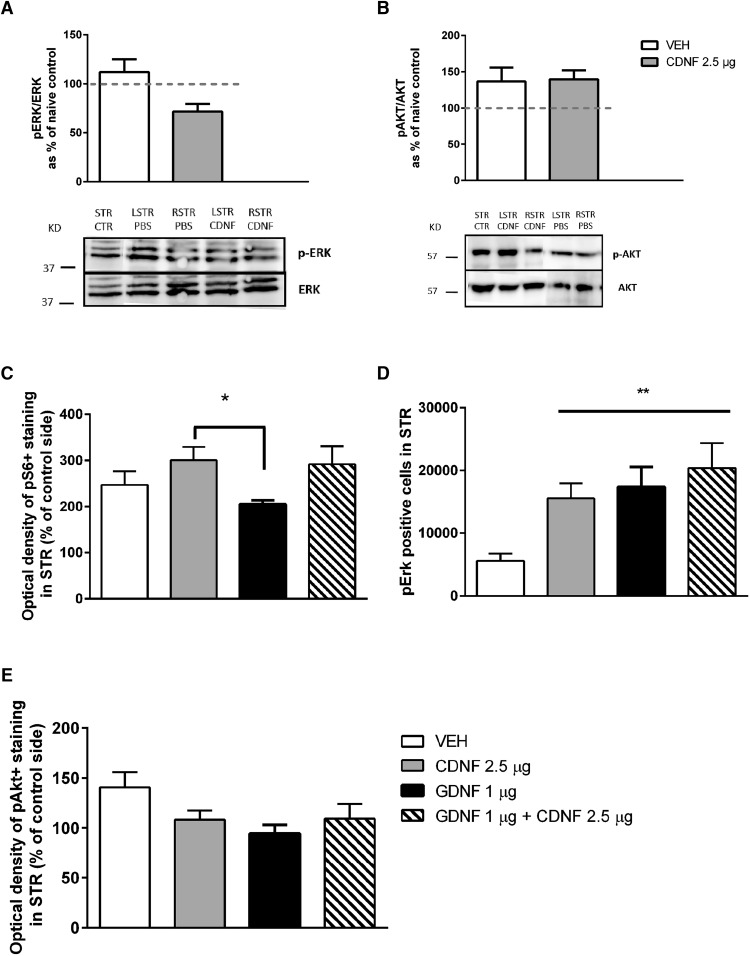
Differences in activation of ERK1/2 and PI3K/AKT pathways after intrastriatal injection of CDNF and GDNF or their combination in 6-OHDA-lesioned rats. When the rats were dissected 4 h after the NTF injection, the MAPK or PI3K/AKT pathway was not activated in 6-OHDA-lesioned rats. CDNF (2.5 µg) did not have significant effect on MAPK (***A***) or PI3K/AKT/mTOR (***B***) pathways. CDNF (2.5 µg) increased ribosomal protein S6 phosphorylation in the STR in comparison to rats treated with GDNF (1 µg) (***C***). The number of pErk-positive cells in STR was increased by all treatments (***D***), while there was no effect on OD of pAkt-positive fibers in STR (E). Tukey/Kramer *post hoc* analysis after one-way ANOVA. *p < 0.05, **p < 0.01.

To study whether CDNF, GDNF, or their combination can activate the MAPK pathway *in vivo*, rats were injected unilaterally with the trophic factors into left STR and PBS to right side. When measured 1 h after NTF injection, GDNF (1 µg) and the combination of GDNF (1 µg) + CDNF (2.5 µg) seemed to activate pERK/ERK pathway, while CDNF alone or PBS (sham lesion) had no effect (Fig. 3*A*). When measured 4 h after NTF injection, CDNF (2.5 µg) did not activate the pERK/ERK pathway after bilateral administration to naïve rats (Fig. 3*C*) or unilateral to the 6-OHDA-lesioned rats (Fig. 4*A*).

Activation of the PI3K-AKT/mTOR pathway is important in the regulation of ER stress, and therefore the striatal samples were also analyzed for pAKT/AKT. One hour after injection, GDNF activated AKT, whereas CDNF or the combination had no effect (Fig. 3*B*). However, when rats were dissected 4 h after the CDNF (2.5 µg) injection, the pAKT/AKT was increased in nonlesioned rats (Fig. 3*D*) and in 6-OHDA-lesioned brain (Fig. 4*B*). In 6-OHDA-lesioned rats also PBS was able to activate the AKT pathway (Fig. 4*B*). These data suggest that CDNF is able to activate the AKT pathway in nonlesioned and in 6-OHDA-lesioned rats in a delayed fashion compared with GDNF-induced activation.

The ability of trophic factors to activate intracellular signaling pathways was studied also when administered four weeks after the 6-OHDA lesion. Phosphorylation of Akt, Erk, and ribosomal protein S6 in STR were analyzed by immunohistochemistry 4 h after the treatments. CDNF increased the phosphorylation of S6 in STR when compared with GDNF. The combination of CDNF + GDNF did not have any additive effect (Fig. 4*C*). CDNF (2.5 µg), GDNF (1 µg), and their combination increased Erk phosphorylation, and there was a trend that the CDNF + GDNF combination activated Erk the most (Fig. 4*D*). None of the treatments [CDNF (2.5 µg), GDNF (1 µg), or their combination] was able to activate Akt when measured four weeks after trophic factor administration (Fig. 4*E*).

### Intracellular ER stress markers

**Figure 5.**
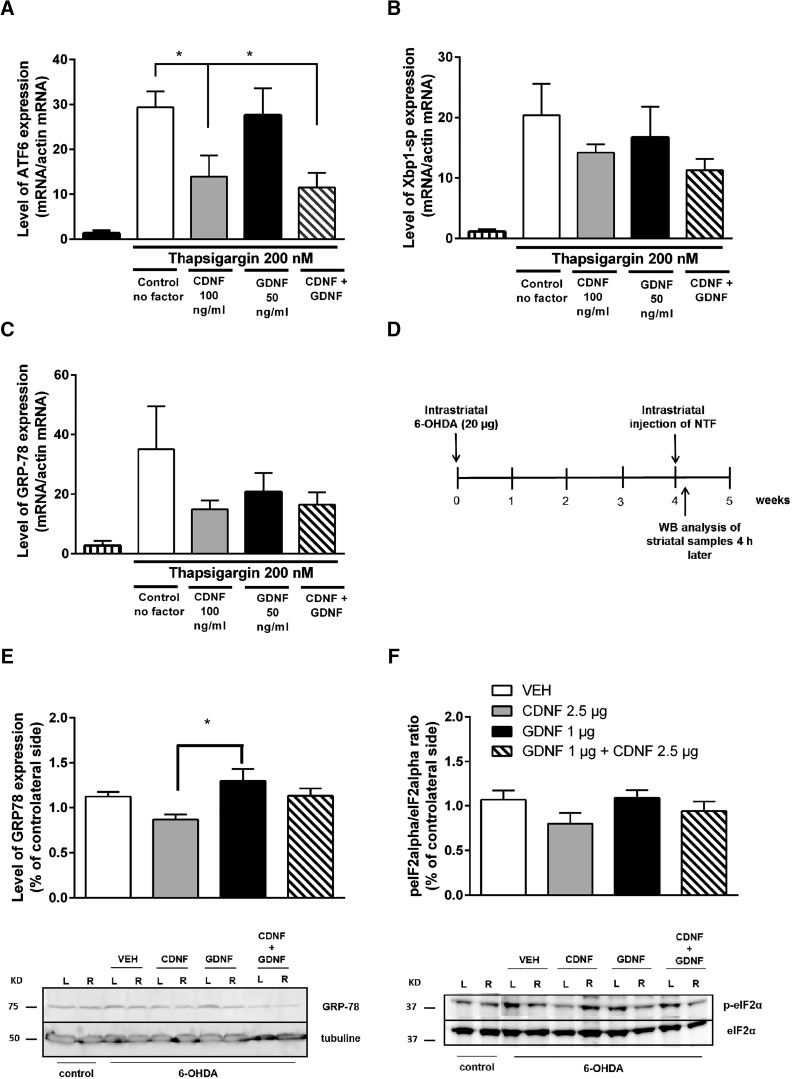
Effects of CDNF and GDNF and their combination on ER stress-triggered UPR markers in cultured DA neurons and in rat 6-OHDA model *in vivo*. ***A*–*C***, E13 dopamine neurons were cultured 5-7 d with GDNF (50 ng/ml). Then, the cultures were treated with thapsigargin (200 nM) to induce ER stress. CDNF (100 ng/ml), GDNF (50 ng/ml), or their combination was added to the cultures at the same time. The expression levels were normalized to the levels of β-actin in the same samples. CDNF and the combination reduced expression of ATF6 mRNA (***A***). Levels of Xbp1-sp (***B***) and GPR-78 (***C***) were not changed. ***D-*-*F***, Effect of CDNF and GDNF on ER stress markers in the rat 6-OHDA model. ***D***, Experimental design for ER stress markers analyses. Rats were administered 6-OHDA (20 µg) unilaterally into the left striatum (L). Four weeks later, the rats received a single injection of either vehicle (PBS), CDNF (2.5 µg), GDNF (1 µg), or their combination into the lesioned striatum. Rats were killed 4 h after NTF injection, and striata were dissected for Western blot analyses. ***E***, Injection of CDNF decreases GRP78 protein levels in striata of 6-OHDA-lesioned rats compared with PBS (*p* = 0.2) or GDNF treatment (*p* < 0.05). The level of expression is expressed as % of intact contralateral site of the brain. Representative Western blot images of GRP78 expression. Control shows basal level of GRP78 in a naïve rat brain. β-tubulin is used as loading control. ***F***, CDNF-treated animals showed a decrease in the activation of P-eIF2α compared with the vehicle-treated rats. Representative Western blot images of P-eIF2α and total eIF2α. Control shows basal level of P-eIF2α and total eIF2α in a naïve rat brain. Mean ± SEM. *n* = 5-6 in each group. Tukey/Kramer *post hoc* analysis after one-way ANOVA. **p < 0.01.

Effects of CDNF and GDNF and their combination on ER stress-triggered UPR markers were studied in cultured embryonic dopamine neurons after induction of ER stress by thapsigargin. The results are disclosed as a percentage of the number of neurons counted after plating of primary neurons. CDNF and the combination of CDNF and GDNF was able to reduce ER tress marker ATF6 mRNA levels, whereas GDNF alone had no effect (Fig. 5*A*). CDNF also showed tendency in reducing Xbp1-sp mRNA levels (Fig. 5*B*) and GRP78 (alias BiP) more than GDNF treatment bringing out differences between CDNF and GDNF signaling (Fig. 5*A–C*).

In hemiparkinsonian rats, the effects of CDNF and GDNF on the levels of the ER stress-triggered UPR markers were analyzed at four weeks following the 6-OHDA lesion. Four hours later, rats were killed, and striatal samples were dissected (Fig. 5*D*). GRP78 protein levels and phosphorylation of eIF2α (p-eIF2α/eIF2α) were analyzed by Western blotting.

CDNF-treated animals showed reduced expression of GRP78 when compared with vehicle- and GDNF-treated animals (x = *F*_(3,21)_ = 3.973, *p* = 0.025; *n* = 8 per group). Tukey *post hoc* test after One-way ANOVA revealed differences between CDNF (2.5 µg) and GDNF (1 µg) groups (*p* < 0.014; Table 1, i). The combination of GDNF and CDNF did not show any effect (Fig. 5*E*,*F*; Table 1, j and k). CDNF, but not GDNF, nor the combination of the NTFs, decreased the phosphorylation of eIF2α when compared with the vehicle-treated group, although the difference is not statistically significant (Fig. 5*F*; Table 1, l and m).

### Effect of CDNF, GDNF, and their combination on TH- and DAT protein levels in 6-OHDA-lesioned and sham-lesioned brain

#### Western blotting

**Figure 6.**
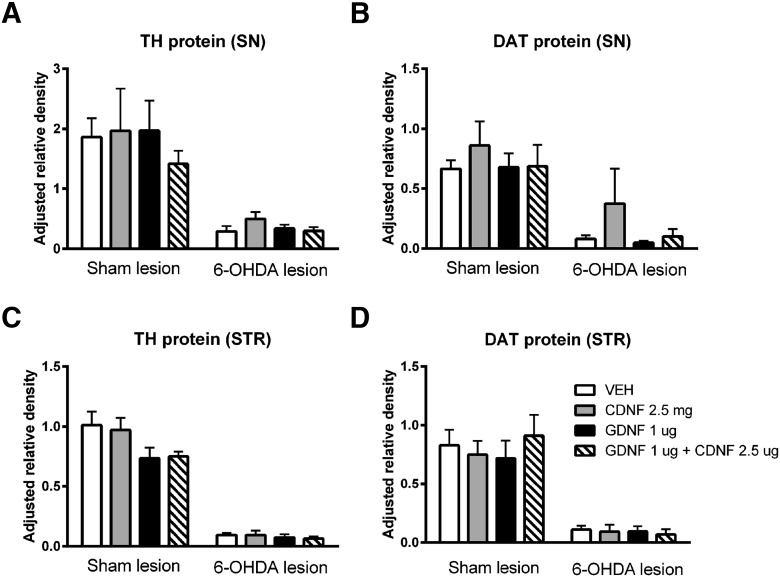
Effect of CDNF, GDNF, and their combination on TH- and DAT protein levels in 6-OHDA-lesioned and sham-lesioned brain. Four weeks after lesioning, sham-lesioned or 6-OHDA-lesioned rats received an intrastriatal injection of vehicle (VEH), CDNF, GDNF, or a combination of CDNF and GDNF. Eight weeks after lesioning, rat SN and striatum (STR) protein expression were analyzed from total protein samples (13 µg for SN samples and 15 µg for STR samples, respectively) using Western blotting. 6-OHDA lesion resulted in a marked decrease in both TH and DAT protein levels in the SN (***A***, ***B***) and STR (***C***, ***D***). None of the treatments were able to significantly affect the TH and DAT protein levels after sham or 6-OHDA lesion, although GDNF alone or in combination with CDNF tended to decrease the levels of TH in sham-lesioned STR (***C***). Results are shown as the group mean ± SEM of protein band density relative to the density of a control sample (pooled from four naive STR or SN) and β-actin. *n* = 3-5 in each group. Tukey/Kramer *post hoc* analysis after one-way ANOVA. TH-levels in SN (***A***) and striatum (***C***) and DAT-levels in SN (***B***) and STR (***D***) did not show statistically significant changes.

None of the NTF treatments caused significant changes in DAT or TH protein levels in SN (Fig. 6*A*,*B*) or striatum (Fig. 6*C*,*D*) of sham- or 6-OHDA-lesioned rats. GDNF, both alone or in combination with CDNF, lead to a small decrease in striatal levels of TH (Fig. 6*C*), while CDNF alone seemed to have a small increasing effect on the DAT levels in the lesioned SN (Fig. 6*D*). However, none of these changes were statistically significant.

## Discussion

CDNF and GDNF have similar neurotrophic effects on 6-OHDA-lesioned DAergic neurons *in vivo* (Lindholm et al., 2007; Lindholm and Saarma, 2010; Voutilainen et al., 2011) despite their different amino acid sequences and 3D structures. Both are secreted proteins, but CDNF is also an ER resident protein and may regulate, similarly to the homologous MANF protein (Apostolou et al., 2008; Lindahl et al., 2014), ER stress inside the cells. Therefore, we wanted to test whether a combination of submaximal doses of CDNF and GDNF would have an enhanced effect. Also, the different expression pattern of GDNF (Stromberg et al., 1993; Suvanto et al., 1996; Trupp et al., 1997; Nosrat et al., 1997; Golden, et al., 1998) and CDNF (Lindholm et al., 2007) in rodent brain prompted these studies. Indeed, a combination of 2.5 µg of CDNF and 1 µg of GDNF was more effective than either protein alone in restoring DAergic function in the unilateral 6-OHDA rat PD model. Moreover, the two NTFs appeared to exert *in vivo* effects at least partially via different mechanisms: CDNF may have a dual action via activation of PI3K/Akt pathway and down-regulation of intracellular ER stress-induced protein GRP78 and decreasing eIF2α phosphorylation, while GDNF was activating ERK1/2 and Akt pathways but did not change the levels or phosphorylation of ER stress-related protein or ER stress protein mRNA levels.

Additive or synergistic effects of NTFs are not well established despite the potential importance of such applications for NTF based therapies of PD and other neurodegenerative diseases. *In vitro*, combinations of GDNF with other NTFs such as BDNF and ciliary NTF (CNTF) (Zurn et al., 1996) and cardiotrophin-1 (CT-1) (Arce et al., 1998) have shown to improve motoneuron survival and differentiation. It should be noted that CNTF and CT-1 act differently than GDNF activating completely different receptor systems. In cultured rat fetal nigral tissue coexposure to GDNF and neurotrophin 4/5 was beneficial for DAergic cell survival and functionally related biochemical parameters (Meyer et al., 2001). In these neurons also the effects of GDNF and pleiotrophin (alias HB-GAM) are additive (Hida et al., 2003). *In vivo* settings with combined administration of neurturin or GDNF with BDNF had additive effects on the survival of axotomized retinal ganglion cells, suggesting that these NTFs act independently to rescue injured cells (Koeberle and Ball, 2002). Interestingly, neuronal survival and signaling mediated by GDNF was shown to require the presence of transforming growth factor (TGF)-β (Peterziel and Unsicker, 2002; Schober et al., 2007). TGF-β regulates the availability of the glycosyl phosphatidylinositol GPI-anchored GFRα1 by promoting the recruitment of the receptor to the plasma membrane (Peterziel et al., 2007). Also, additive effects of GDNF with TGF-β1, given by osmotic minipumps to the dorsal striatum (Gonzalez-Aparicio et al., 2010), and of MANF with CDNF, given as a special lentiviral gene construct to the SN (Cordero-Llana et al., 2015), were reported in the 6-OHDA model of PD in rats. The latter finding suggests that although MANF and CDNF belong to the same protein family, even these proteins may differ in their mode of action. Finally, Dodge et al. (2010) reported that no additive effect was seen in a genetic SOD1 model of ALS when IGF-1 and VEGF-165-expressing vectors were given together suggesting that these growth factors act on similar signaling pathways (Dodge et al., 2010).

An intrastriatal injection of 6-OHDA reduced protein levels of TH and DAT, and none of the growth factor treatments were able to significantly restore the expression of these dopaminergic cell markers. Administration of CDNF into intact brain did not have any effects on the levels of DAT and TH. This is in line with our previous studies, where no change in the number of TH-expressing neurons was seen after a two-week infusion of CDNF (Voutilainen et al., 2011) or after a single CDNF injection (Airavaara et al., 2011). GDNF, on the other hand, has been shown to have pronounced effects on the intact dopaminergic system, including changes in TH levels and sprouting of TH-reactive fibers, as well as increase in dopamine turnover and stimulus-induced dopamine release (Hebert et al., 1996; Rosenblad et al., 2003; Salvatore et al., 2004; Voutilainen et al., 2011). In our present study, this is reflected as a (statistically nonsignificant) decrease in nigral and striatal levels of TH protein. However, in stereological cell count, the combination of CDNF and GDNF was able to increase the number of TH-positive cells in SNpc, and all treatments increased the number of Nissl-positive cells in 6-OHDA-treated animals.

Different mechanisms of action of GDNF and CDNF could, in fact, be predicted from their distinct 3D structures and cellular localization (Mätlik et al., 2015; Voutilainen et al., 2015). The GDNF monomer is composed of two long fingers formed by pairs of antiparallel β-strands connected by loops, a cystine-knot core motif, and an α-helical heel region. The two GDNF monomers are arranged in a head-to-tail orientation to form a homodimer, in which the two helices from the heel region flanking two cystine-knot motifs are at the center (Parkash and Goldman, 2009). GDNF preferentially binds to the GFRα1 coreceptor, and then GDNF-GFRα1 complex interacts with receptor tyrosine kinase RET triggering its dimerization, kinase activation and phosphorylation at RET intracellular tyrosine residues. Further actions in neurons go through the MEK/ERK, SRC, PLCγ, and PI3K/AKT pathways (McAlhany et al., 2000; Airaksinen and Saarma, 2002; Villegas et al., 2006). GDNF can bind to the neural cell adhesion molecule (NCAM)-GFRα1 complex and activate the downstream targets Src-type kinases FYN/FAK in regulating axonal guidance and corneal regeneration (You et al., 2001; Paratcha et al., 2003; Euteneuer et al., 2013). By binding to heparin sulphate proteoglycan syndecan-3 GDNF can induce neurite outgrowth and migration of cortical neurons (Bespalov et al., 2011).

CDNF (like MANF) consists of two domains (Parkash and Goldman, 2009; Hellman et al., 2011, Latge et al., 2015). The amino-terminal domain of CDNF is a saposin-like lipid-binding domain, which suggests that CDNF may bind lipids or membranes (Parkash and Goldman, 2009). Based on the solution NMR-structure studies, the carboxy-terminal domain of MANF, and CDNF, resembles the SAP domain of Ku-70 that has an antiapoptotic function in the cytoplasm (Hellman et al., 2011; Latge et al.; 2015). Data from our laboratory (Hellman et al., 2011; Lindahl et al., 2014) and others (Glembotski et al., 2012) indicate that similar to MANF, the mechanism of action of CDNF is critically associated with the regulation of ER stress. Indeed, our present data are in line with those of Glembotski et al. (2012), indicating that CDNF may interact with chaperon GRP78. This conclusion was further supported by our finding that CDNF was able to inhibit expression of GRP78 and ATF6 in primary cultured DA neurons under thapsigargin-induced ER-stress. Furthermore, CDNF, but not GDNF, decreased the phosphorylation of eIF2α, indicating that CDNF may exert its trophic effect by regulating the ER stress response in the dying neurons of the 6-OHDA-lesioned brains. Thus, the mode of action of CDNF mainly operating in the ER and by activating the PI3K-Akt pathway-promoting cell survival would be drastically different from that of GDNF (K. Krieglstein and M. Saarma, personal communication). These data support our previous observations that CDNF activates PI3K-Akt pathway in ER-stressed DA neurons (K. Krieglstein and M. Saarma, personal communication). Here, we show that CDNF can activate the same pathway *in vivo* in the midbrain.

GDNF and the combination of GDNF and CDNF, but not CDNF alone, activated mainly the pERK1/pERK2 pathway. When the rats were dissected 4 h after CDNF injection, CDNF preferably activated AKT pathway both in the lesioned and nonlesioned rat brains. Indeed, CDNF increased expression of pS6 in striatum significantly as compared with GDNF.

The observations of this study may have implications for future clinical studies were combination of CDNF and GDNF (or other combinations) may be evaluated in the treatment of degenerative brain diseases. Since oxidative ER stress is an important pathway to cell death in neurodegenerative diseases (Ryu et al., 2002), CDNF may limit disease progression. In addition, recent studies suggest that CDNF may support neuroregeneration also by suppressing neuroinflammation via its effects on astrocytes and microglial cells (Nadella et al., 2014). Furthermore, there is evidence that CDNF can dissolve α-synuclein aggregates in neurons Latge et al. (2015). These characteristics may be of particular importance as volume of distribution of CDNF in the brain is much bigger than that of GDNF (Voutilainen et al., 2011).

This is the first *in vivo* study to show the additive effect of CDNF and GDNF in the rat 6-OHDA model of PD. This is also the first study to show that CDNF activates PI3K-Akt pathways *in vivo* and regulates ER stress markers GRP78 and eIF2a both *in vitro* and *in vivo*. CDNF reduced the upregulation and/or phosphorylation of ER stress-response proteins in DA neurons exposed to thapsigargin *in vitro*. These results indicate that the effects of CDNF on ER stress contribute to the neurorestorative effect of CDNF alone and in combination with GDNF in 6-OHDA induced PD model. Since submaximal doses of two proteins, CDNF and GDNF, lead to full effect, less NTF is needed, less side effects will likely occur and possible drug therapy for degenerative diseases will be cheaper. Further studies are needed to demonstrate whether CDNF can rescue DA neurons in an α-synuclein model of PD and whether CDNF would work in other neurodegenerative models where ER stress is present.

## Synthesis

Reviewing Editor: Jörg Schulz, RWTH Aachen University Decisions are customarily a result of the Reviewing Editor and the peer reviewers coming together and discussing their recommendations until a consensus is reached. When revisions are invited, a fact-based synthesis statement explaining their decision and outlining what is needed to prepare a revision will be listed below. The following reviewer(s) agreed to reveal their identity: Edgar Kramer, Björn Spittau.

The authors addressed all issues that were raised, which resulted in a significant improvement of the manuscript.

There are only some minor comments left that should be addressed:

1) The first sentence in the introduction is difficult to understand und should be modified.

2) Several references seem not properly formatted such as Garea-Rodriquez et al., 2016; Neves; Suvanto et al., 2006.

3) Please close bracket in line 296 after Fig.5B.

4) Please label X-axis of Fig.1D as in Fig. 1B and C for easy comparison.
